# Comparison of the Chemical Compositions of the Cuticle and Dufour’s Gland of Two Solitary Bee Species from Laboratory and Field Conditions

**DOI:** 10.1007/s10886-017-0844-x

**Published:** 2017-05-12

**Authors:** Theresa L. Pitts-Singer, Marcia M. Hagen, Bryan R. Helm, Steven Highland, James S. Buckner, William P. Kemp

**Affiliations:** 1 USDA ARS Pollinating Insects Research Unit, Utah State University, Logan, UT 84322 USA; 2 USDA ARS Biosciences Research Laboratory, Red River Valley Agricultural Research Center, Fargo, ND 58102 USA; 3 Department of Biological Sciences, North Dakota State University, Fargo, ND 58108 USA; 4 Bureau of Land Management - Mt. Lewis Field Office, 50 Bastian Rd., Battle Mountain, NV 89820 USA

**Keywords:** Lipids, Megachilidae, *Megachile rotundata*, Nest-marking, Nest recognition, *Osmia lignaria*

## Abstract

Species-specific biochemistry, morphology, and function of the Dufour’s gland have been investigated for social bees and some non-social bee families. Most of the solitary bees previously examined are ground-nesting bees that use Dufour’s gland secretions to line brood chambers. This study examines the chemistry of the cuticle and Dufour’s gland of cavity-nesting *Megachile rotundata* and *Osmia lignaria*, which are species managed for crop pollination. Glandular and cuticular lipid compositions were characterized and compared to each other and according to the nesting experience of adult females. Major lipid classes found were hydrocarbons, free fatty acids, and wax esters. Many components were common to the cuticle and Dufour’s glands of each species, yet not identical in number or relative composition. Wax esters and fatty acids were more prevalent in Dufour’s glands of *M. rotundata* than on cuticles. Wax esters were more abundant on cuticles of *O. lignaria* than in Dufour’s glands. In both species, fatty acids were more prevalent in glands of field-collected females compared to any other sample type. Chemical profiles of cuticles and glands were distinct from each other, and, for *O. lignaria*, profiles of laboratory-maintained bees could be distinguished from those of field-collected bees. Comparison of percentiles of individual components of cuticular and glandular profiles of the same bee showed that the proportions of some cuticular components were predictive of the proportion of the same glandular components, especially for nesting females. Lastly, evidence suggested that Dufour’s gland is the major source of nest-marking substances in *M. rotundata*, but evidence for this role in *O. lignaria* was less conclusive.

**Electronic supplementary material:**

The online version of this article (doi:10.1007/s10886-017-0844-x) contains supplementary material, which is available to authorized users.

## Introduction

The Dufour’s gland is an exocrine gland associated with the sting apparatus in all aculeate hymenoptera. Its function in communication has been demonstrated or implicated in many hymenoptera, such as laying down trail and recruitment pheromones in ants and providing nest recognition and mating status for many social wasps and bees (Abdalla and da Cruz-Landim 2001a, b, c, d; Billen 1987; Duffield et al. 1984; Mitra 2013). For non-social bees, Dufour’s gland secretions serve primarily as nest cell linings (Cane 1981; Mitra 2013), although they may also function to maintain humidity, defend against microbial infection, and even serve as a food source (Duffield et al. 1984; Hefetz 1998; Mitra 2013). Most of the solitary bees studied thus far are ground-nesters that use Dufour’s gland secretions to line brood chambers (Hefetz 1998). Species studied represent Colletidae, Halictidae, Andrenidae, Mellitidae, and the apid tribes Anthophorini, Eucerini, and Xylocopini (Albans et al. 1980; Cane 1983; Duffield et al. 1984; Hefetz 1998; Mitra 2013; Tengö and Bergström 1975; Tengö et al. 1985, 1991, 1992; Vinson et al. 1978). However, the functional, morphological and chemical characteristics of the typically conspicuous Dufour’s glands of many of the world’s 16,000+ solitary bee species (Michener 2000) remain speculative or unknown (Billen and Morgan 1998). Of particular interest is the possible role of the Dufour’s gland in solitary bee communication, especially in the unique marking of nests with individual and distinguishable recognition cues.

In addition to the Dufour’s and other exocrine glands (especially in social hymenoptera), cuticular lipids play a role in communication. The primary function of the insect cuticle is to serve as a moisture barrier to prevent desiccation and protect the body from intrusion by foreign substances and as a layer that provides a physical framework that includes areas for muscle attachment (Blomquist et al. 1998). However, cuticular lipids further serve secondary functions as inter- and intraspecific communication cues including recognition between conspecifics, sexes, and nestmates in social colonies (Blomquist et al. 1998; Singer 1998). Cuticular lipids of solitary bees may also serve communicative roles, as indicated by the attraction of *M. rotundata* males to solvent extracts of the cuticles of young females (Paulmier et al. 1999).

Two agriculturally important, managed solitary bees in the United States are *M. rotundata* L. and *O. lignaria* Say (Hymenoptera: Megachilidae)*.* Understanding chemical-mediation of the nesting behavior of these cavity-nesting species may have management implications. Alfalfa seed production is largely dependent on *M. rotundata* for seed set (Pitts-Singer and Cane 2011), and the use of *O. lignaria* for pollination of some fruit and nut trees is increasing (Bosch and Kemp 2001; Peterson and Artz 2014). Females of both species build and lay eggs in their own nests in existing cavities. They readily live in large aggregations, a characteristic that facilitates their use of artificial cavities provided for them in agricultural settings. However, where they are used for crop pollination in the U.S., the retention of females at nest sites is far less than the number of bees released at or near those sites (Artz et al. 2013, 2014; Bosch and Kemp 2001; Pitts-Singer 2013; Tepedino and Frolich 1982; Torchio 1982). The choice of individual nest cavities for establishing a new nest is attributed to attraction of nest-seeking females to odors from previously-used nests, which are preferred over those in which bees have never nested (Buttery et al. 1981; Parker et al. 1983; Pitts-Singer 2007; Stanley and Pitts-Singer 2011). Finding the attractive compounds in those old nests may help to retain bees in commercial settings, as has recently been done for *O. lignaria* (Pitts-Singer et al. 2016).

However, once females select a nesting cavity where many cavities are provided in close proximity, they rely on other cues to find their own nests after foraging bouts. Long-range cues are visual, while short-range cues are olfactory (Guédot et al. 2006, 2007, 2013; Steinmann 1973). At times, bees are visually misguided and attempt to enter cavities belonging to conspecifics. When this occurs, the female detects an unfamiliar odor that may be a deterrent because it indicates residency by another bee; therefore, the bees immediately back out of any foreign cavity. In manipulative experiments, whole or sections of glass tube nests being used by actively nesting *M. rotundata* and *O. lignaria* females were removed and replaced with new, unused tubes while the foraging females were away. Upon returning to the nest sites, these females had difficulty finding their own nests and reluctantly reentered them (Steinmann 1973; Guédot et al. 2006, 2013). Guédot et al. (2006, 2013) also observed female bees inside their nests displaying body movements and positions consistent with employing the abdomen for nest-marking and concluded that replacement of the used tubes had removed discriminating, individual recognition cues that were applied intentionally by the bees. The definitive source of the chemical cues was not revealed in that study, but was suspected to be lipids from the cuticle, the Dufour’s gland, or both.

Although the Dufour’s gland of some megachilid species has been examined, no studies have been performed for the commercially available species. The cuticular lipids of just-emerged as well as nesting *M. rotundata* and *O. lignaria* females have been reported (Buckner et al. 2009; Guédot et al. 2006, 2013), but how a female’s cuticular lipids compare to her Dufour’s gland content is unknown. The contents of the Dufour’s gland of four ground-nesting and two cavity-nesting *Megachile* species contain triglycerides with 2–16-carbon fatty acids (Cane and Carlson 1984; Williams et al. 1986), but it cannot be assumed that the Dufour’s glands of all bees share common chemistry, nor that all analytical chemistry is performed using identical techniques with similar levels of detection and sensitivity. The Dufour’s gland structure was recently described for *M. rotundata* and *O. lignaria* (Pitts-Singer et al. 2012), and has previously been described for *O. cornifrons* (Radoszkowski) (Barrows et al. 1986), yet the Dufour’s gland contents of these bees were not examined. If the passive or intentional smearing of cuticular and/or Dufour’s components onto the inner walls of solitary bee nest tunnels provides a unique nest recognition cue, then the lipids of nesting females need to be examined.

This study was performed to identify and compare the lipid profiles of cuticles and Dufour’s glands of *M. rotundata* and *O. lignaria*. Within species, the lipid compositions of cuticles and glands of laboratory-reared, non-nesting females and those of females from the field that had been reproductively active also were compared to determine if the nesting environment and physiological state of females influence the composition of their cuticles and glands. The distinction between chemical profiles of all sample types was determined. Lastly, we discuss the possibility that compounds of the cuticle and Dufour’s gland have compositions indicative of an individual bee that exemplifies the uniqueness of a female’s chemical signal and her nest-marking cues.

## Methods and Materials

### Sample Collection and Lipid Extraction

Samples of both *M. rotundata* and *O. lignaria* were collected over several years (Supplementary Table 1). All laboratory bees were from stocks managed at USDA ARS Pollinating Insects Research Unit (PIRU) that were warmed in incubators until adult emergence using standard protocols (Bosch and Kemp 2001; Richards 1984). Laboratory-reared *M. rotundata* were purchased from Saskatchewan, Canada, and *O. lignaria* were obtained from a “bee-trapper” who collects bees from northern Utah, USA. To maintain emerged bees in the laboratory, females were kept with males in benchtop Plexiglas boxes and fed 10% honey-water (except in 2006 when *M. rotundata* were given 25% sucrose-water solution) (Pitts-Singer 2007). The laboratory bees were freeze-killed at 2–7 days old (Supplementary Table 1).

**Table 1:** The average percentage composition (± SE) of compounds in lipid extracts of the cuticles and Dufour’s glands from field-collected (*N* = 10) and laboratory-reared (*N* = 12) *Megachile rotundata* females

Peak^b^	Compound	Mean percent composition^a^ ± SE			
		Cuticle		Dufour’s Gland	
		Field	Laboratory	Field	Laboratory
1	14:0 FFA^c^	0.3 ± 0.1	–	3.2 ± 0.6	1.1 ± 0.5
2	14:0 FAEE	–	t	–	t
3	16:0 FFA	1.4 ± 0.3	0.6 ± 0.1	13.1 ± 1.4	7.2 ± 1.6
4	18:0 WE	0.1 ± 0.1	–	t	0.1 ± 0.1
5	16:0 FAEE	t	t	t	0.3 ± 0.1
6	16:0 FAIPE	t	–	0.7 ± 0.1	–
7	18 ALD	–	0.2 ± 0.0	–	–
8	21:0 HC	t	t	–	0.1 ± 0.1
9	18:U^d^ FFA	1.0 ± 0.2	0.1 ± 0.1	16.1 ± 2.2	5.3 ± 1.6
10	18:0 FFA	0.9 ± 0.2	t	10.3 ± 1.3	4.4 ± 1.5
11	18:U FAEE	0.4 ± 0.1	0.8 ± 0.2	2.6 ± 0.6	4.5 ± 1.5
12	20:0 WE	0.1 ± 0.1	t	0.3 ± 0.3	0.5 ± 0.3
13	18:0 FAEE	0.2 ± 0.1	0.2 ± 0.0	1.1 ± 0.2	1.1 ± 0.3
14	18:0-OAc	0.4 ± 0.1	0.4 ± 0.1	3.4 ± 1.1	3.6 ± 0.6
15	22:0 HC	t	t	t	–
16	20 ALD	–	0.2 ± 0.0	–	–
17	23:1 HC^e^	0.3 ± 0.0	0.5 ± 0.0	t	0.4 ± 0.1
18	23:0 HC	1.5 ± 0.2	2.6 ± 0.2	0.7 ± 0.1	1.9 ± 0.2
19	20:1 FFA	0.1 ± 0.1	0.1 ± 0.1	0.7 ± 0.3	0.5 ± 0.4
20	20:1 FAEE	t	0.1 ± 0.1	0.2 ± 0.1	0.6 ± 0.4
21	20:0 FFA	0.1 ± 0.1	–	–	-
22	22:0 WE	0.1 ± 0.1	t	0.2 ± 0.1	0.1 ± 0.1
23	20:1-OAc	t	t	1.0 ± 0.5	0.8 ± 0.3
24	18–4:0	–	t	–	0.6 ± 0.2
25	24:1 HC	t	0.4 ± 0.0	t	t
26	20:0 FAEE	t	–	–	0.2 ± 0.1
27	20:0-OAc	t	t	–	1.1 ± 0.8
28	24:0 HC	0.3 ± 0.0	0.3 ± 0.0	t	0.2 ± 0.1
29	Unknown	–	–	1.6 ± 0.4	0.7 ± 0.4
30	22 ALD	t	0.2 ± 0.0	–	–
31	25:1 HC	24.1 ± 1.7	34.1 ± 1.0	7.0 ± 1.1	17.9 ± 2.8
32	25:0 HC	14.1 ± 0.7	16.9 ± 0.5	2.8 ± 0.7	9.0 ± 1.3
33	25A HC	t	t	–	–
34	20:1–4:0	t	t	–	0.2 ± 0.1
35	22:1 FAEE	–	t	–	0.2 ± 0.1
36	24:0 WE	t	t	0.5 ± 0.1	0.3 ± 0.1
37	20:0–4:0	–	t	–	0.2 ± 0.1
38	26:1 HC	0.8 ± 0.0	0.7 ± 0.0	–	t
39	26:0 HC	0.4 ± 0.0	0.3 ± 0.0	0.3 ± 0.1	0.2 ± 0.0
40	24 ALD	t	t	–	–
41	11-oxo-pentacosane	t	t	t	0.1 ± 0.1
42	9-oxo-pentacosane	t	t	t	t
43	27:1 HC	16.5 ± 1.6	14.1 ± 0.6	3.5 ± 0.5	5.2 ± 0.7
44	27:0 HC	9.1 ± 0.2	7.4 ± 0.3	1.7 ± 0.3	4.2 ± 0.9
45	26:U WE	0.7 ± 0.2	0.6 ± 0.1	5.1 ± 0.8	3.5 ± 1.0
46	27A HC	t	t	–	–
47	26:0 WE	0.8 ± 0.3	0.2 ± 0.0	3.3 ± 1.0	1.5 ± 0.3
48	28:1 HC	t	t	–	-
49	28:0 HC	0.3 ± 0.0	0.2 ± 0.0	0.2 ± 0.1	0.1 ± 0.1
50	26 ALD	t	t	–	–
51	29:1 HC	2.6 ± 0.2	1.9 ± 0.1	0.3 ± 0.1	0.7 ± 0.2
52	29:0 HC	6.4 ± 0.2	3.6 ± 0.2	1.4 ± 0.2	2.8 ± 0.6
53	28:U WE	0.6 ± 0.2	0.4 ± 0.1	2.9 ± 0.8	2.7 ± 0.6
54	29A HC	t	t	–	-
55	28:0 WE	0.3 ± 0.1	0.2 ± 0.1	1.1 ± 0.4	1.6 ± 0.6
56	30:0 HC	0.2 ± 0.0	t	0.3 ± 0.1	t
57	C27 sterol	0.2 ± 0.0	t	1.1 ± 0.2	1.2 ± 0.7
58	31:1 HC	0.6 ± 0.1	0.3 ± 0.0	t	t
59	31:0 HC	3.0 ± 0.2	1.6 ± 0.1	0.9 ± 0.2	1.4 ± 0.3
60	30:U WE	0.6 ± 0.4	0.2 ± 0.1	1.2 ± 0.9	3.9 ± 2.5
61	Unknown	0.3 ± 0.1	0.2 ± 0.0	1.2 ± 0.5	0.9 ± 0.2
62	30:0 WE	0.3 ± 0.1	0.2 ± 0.1	0.9 ± 0.3	1.8 ± 0.6
63	32:0 HC	t	t	0.4 ± 0.2	t
64	30 ALD	t	–	–	–
65	C29 Sterol	0.4 ± 0.0	t	0.8 ± 0.4	0.2 ± 0.1
66	33:1 HC	0.2 ± 0.1	t	t	0.1 ± 0.1
67	33:0 HC	0.7 ± 0.1	0.4 ± 0.0	0.6 ± 0.3	0.3 ± 0.1
68	32:1 WE	0.2 ± 0.1	t	0.7 ± 0.3	0.8 ± 0.2
69	33A HC	t	t	–	–
70	32:0 WE	t	t	0.8 ± 0.1	0.8 ± 0.1
71	35:1 HC	t	–	–	–
72	35:0 HC	0.2 ± 0.0	t	–	t
73	34:U WE	t	t	1.0 ± 0.4	0.7 ± 0.3
74	35A HC	t	0.0 ± 0.0	–	–
75	34:0 WE	t	t	0.7 ± 0.1	0.6 ± 0.1
76	37:0 HC	t	–	t	–
77	36:U WE	0.5 ± 0.0	0.5 ± 0.1	1.3 ± 0.7	0.8 ± 0.4
78	36:0 WE	t	t	0.3 ± 0.1	0.2 ± 0.1
79	38:U WE	0.4 ± 0.0	0.6 ± 0.1	1.3 ± 1.1	0.9 ± 0.6
80	38:0 WE	t	t	0.2 ± 0.1	t
81	40:U WE	0.2 ± 0.0	0.3 ± 0.0	0.2 ± 0.2	0.1 ± 0.1
82	40:0 WE	0.2 ± 0.1	t	0.2 ± 0.2	–
83	42:U WE	1.0 ± 0.1	1.7 ± 0.2	–	–
84	42:0 WE	0.2 ± 0.0	t	–	–
85	44:U WE	1.0 ± 0.1	1.4 ± 0.2	–	–
86	44:0 WE	0.2 ± 0.0	t	–	–
87	46:U WE	2.4 ± 0.2	2.5 ± 0.3	–	–
88	46:0 WE	0.1 ± 0.0	t	–	–
89	48:U WE	1.3 ± 0.1	1.2 ± 0.1	–	–
90	48:0 WE	t	–	–	–

^a^Percent composition calculated from the integrated peak area from the GC-FID response as described in Materials and Methods

^b^Peak numbers correspond to those in Fig. 1

^c^Lipid classes were abbreviated as follows: *FFA* free fatty acid, *FAEE* fatty acid ethyl ester, *WE* wax ester, *FAIPE* fatty acid isopropyl ester, *ALD* aldehyde, *HC* hydrocarbon, -*OAc* acetic acid ester of the indicated chain-length alcohol, *X-YY:Z* ester with X carbon chain-length alcohol esterified to YY carbon chain-length acid with Z double bonds. The numbers to the left and the right of the colon for hydrocarbons, free fatty acids, fatty acid esters and wax esters are the total number of carbons and the number of double bonds, respectively

^d^U, A mixture was identified with one, two and/or three points of unsaturation at this chain length

^e^Multiple positional isomers of C23 – C31 mono-alkenes resolve into one to three different peaks in Fig. 1, and the total quantity at each chain length is summed on one line in this table

Field-collected, nesting *M. rotundata* were from an unknown mixed source of bee populations from Utah and Canada that were obtained with permission from an alfalfa seed production farm in northern Utah where bees were nesting in tunnels of bee boards housed in domiciles. These field-collected females could have been ones that had overwintered as prepupae, then been incubated for timing adult emergence with onset of alfalfa bloom. These bees would have been field-released for pollination about four weeks prior to collection. Alternatively, field-collected females could have been 2–3 week-old, summer-emerged females, known as second generation bees (Pitts-Singer 2008). Field-collected, nesting *O. lignaria* had been flying in the PIRU research greenhouse where *Phacelia tanacetifolia* Benth (Boraginaceae) had been planted for forage and where artificial nesting tunnels were present; females were 2–7 days old when freeze-killed. All bees were placed in individual glass vials and frozen (−16 °C) at PIRU within 1 h of collection. Excluding *O. lignaria* collected in 2003, each bee was weighed after thawing and before lipids were extracted.

Initially, cuticular lipids were removed from each field-collected *O. lignaria* by gently swirling in either 10 ml hexane for 1 min (at room temperature) followed by a 5 ml hexane rinse for 20 s (Espelie and Hermann 1990; Guédot et al. 2006; Page et al. 1991) or in 3 ml hexane for 60 s, then a quick rinse in 1 ml hexane, followed by a 30 s extraction in 3 ml chloroform (CHCl_3_) and a quick rinse in 1 ml CHCl_3_. The latter protocol was modified in 2006 (and used for all subsequent cuticular extractions of both bee species) using the same previously-used solvents and timing, but changing volumes to 8 ml and 4 ml for initial extractions and quick rinses, respectively. For each sample, extraction solvents were filtered, all solvent rinses were pooled, and the volume was reduced under vacuum and/or a stream of nitrogen (N_2_) gas. Extracted lipids were stored in CHCl_3_ at −16 °C or −80 °C until analyzed. Although different extraction techniques were used, a General Linear Model (SAS 2002-2012, SAS Institute Inc., Cary, NC, USA) detected no significant differences between techniques in the amount of lipids extracted per bee (*F* = 0.71; *d.f.* = 2; *P* = 0.51). Moreover, a comparison of techniques carried out in 2017 showed that differences between individuals extracted by the same method exceed the differences between groups of bees extracted by the different techniques described above.

Immediately after each whole bee had been extracted in solvent, the Dufour’s gland of the same bee was removed under Ringer’s solution using fine forceps. Initially, glands were extracted by macerating each with an insect pin on a glass depression slide (3.2 mm thickness; Ward’s Science, Rochester, NY) under hexane or CHCl_3_. Starting in 2006, each individual gland was transferred to a small glass, tapered tissue grinder (mortar OD × L = 7 × 32 mm; pestle D × L = 3 × 77 mm) to which was added 100 μl CHCl_3_. The gland was ruptured and homogenized until no recognizable tissue remained. A syringe was used to transfer the homogenate to a 1 ml reaction vial and dried under a stream of N_2_. The homogenizer was rinsed with another 100 μl CHCl_3_, and this solvent was transferred to the same reaction vial and dried. The contents of the dry reaction vial were resuspended in CHCl_3_ for storage at −16 °C prior to chemical analysis.

### Chemical Identification and Quantification

Individual lipid components were separated by capillary gas chromatography (GC), quantified by their flame ionization detector (FID) response, and identified by GC-mass spectrometry (GC-MS) (Guédot et al. 2013). For both species, GC-MS analyses were performed as previously described in Buckner et al. (2009). GC-FID analyses conducted in 2003 on field-collected *O. lignaria* were analyzed using an Alltech AT-1HT capillary column and initial oven temperature of 75 °C (Guédot et al. 2006). GC-FID analyses conducted in 2005 and 2006 on laboratory-reared and field-collected *O. lignaria* as well as laboratory-reared *M. rotundata* were performed using a DB-1MS capillary column and initial oven temperature of 50 °C (Guédot et al. 2013). All subsequent GC-FID analyses (in 2008 and 2009; Supplementary Table 1) were performed with this method except that the third oven temperature ramp was eliminated and the second oven temperature ramp, at 10 °C per min, was extended to a final temperature of 340 °C.

Quantities of all alkanes, acetate esters of fatty alcohols and wax esters (WEs) were determined using the integrated peak area data from the FID response to increasing quantities (0.39–200 ng) of the authentic standards, *n*-octacosane (28:0 HC) (Analabs, Inc., North Haven, CT, USA), tricosanyl acetate (23:0-OAc), and tricosanyl heptadecanoate (40:0 WE), respectively. The latter two standards were synthesized according to Nelson et al. (1990). Quantities of alkenes were determined using *n*-octacosane after applying a response factor of 0.9 (Buckner et al. 2009). Quantities of ethyl and isopropyl esters of fatty acids were determined using the authentic standard, methyl heptadecanoate, after first applying a response factor of 1.1 and 0.9, respectively. The FID response of seven authentic free fatty acid (FFA) standards including 14:0, 16:0, 18:0, 16:1, 18:1, 18:2 and 18:3 were compared to the FID signal produced by the same mass of tricosanyl acetate. The FID signal produced by these FFAs consistently averaged 90% of the signal produced by tricosanyl acetate for samples greater than 20 ng. For samples between 1.5 to 20 ng, the relative response of the FFAs decreased with their concentration in a consistent but non-linear manner. As a result, we applied a response factor that varied linearly from 0.19 to 0.26 for FFA peaks less than 1.5 ng, then increased linearly to 0.5 for peaks less than 4.6 ng, and finally increased linearly from 0.9 up to 20 ng. Quantities of aldehydes, butyric acid esters of fatty alcohols, sterols, terpenoids, and unknowns were quantified using the *n*-octacosane standard (Guédot et al. 2013).

**Fig. 1:**
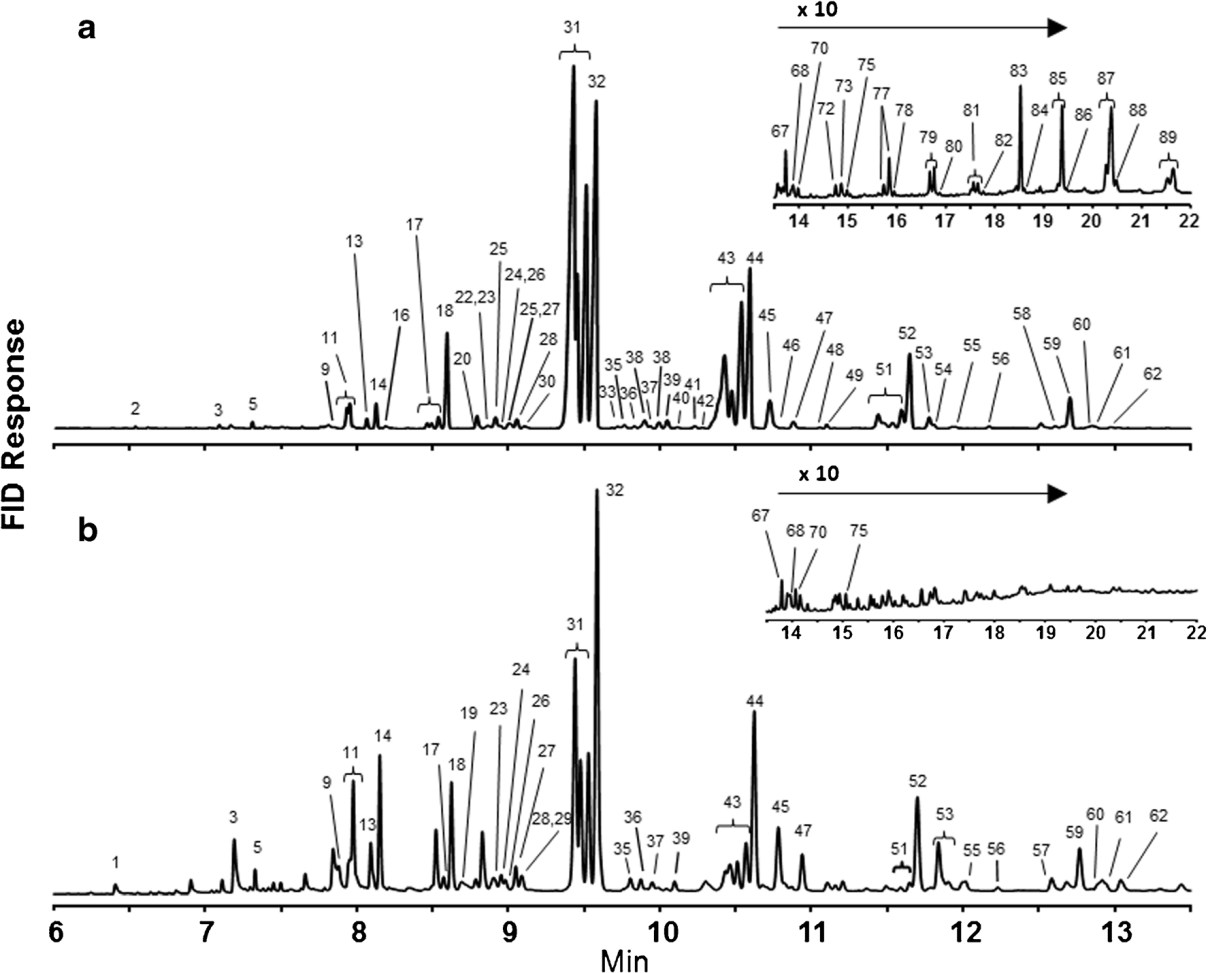
Gas chromatography (GC) flame ionization detector (FID) results of analyses of extracts from the (**a)** cuticle and (**b**) Dufour’s gland of one representative *Megachile rotundata* female. Identifications of known, numbered peaks are reported in Table 1. Noticeable GC peaks that are unlabeled were of insufficient concentration to create a peak in the GC-MS chromatogram

**Fig. 2:**
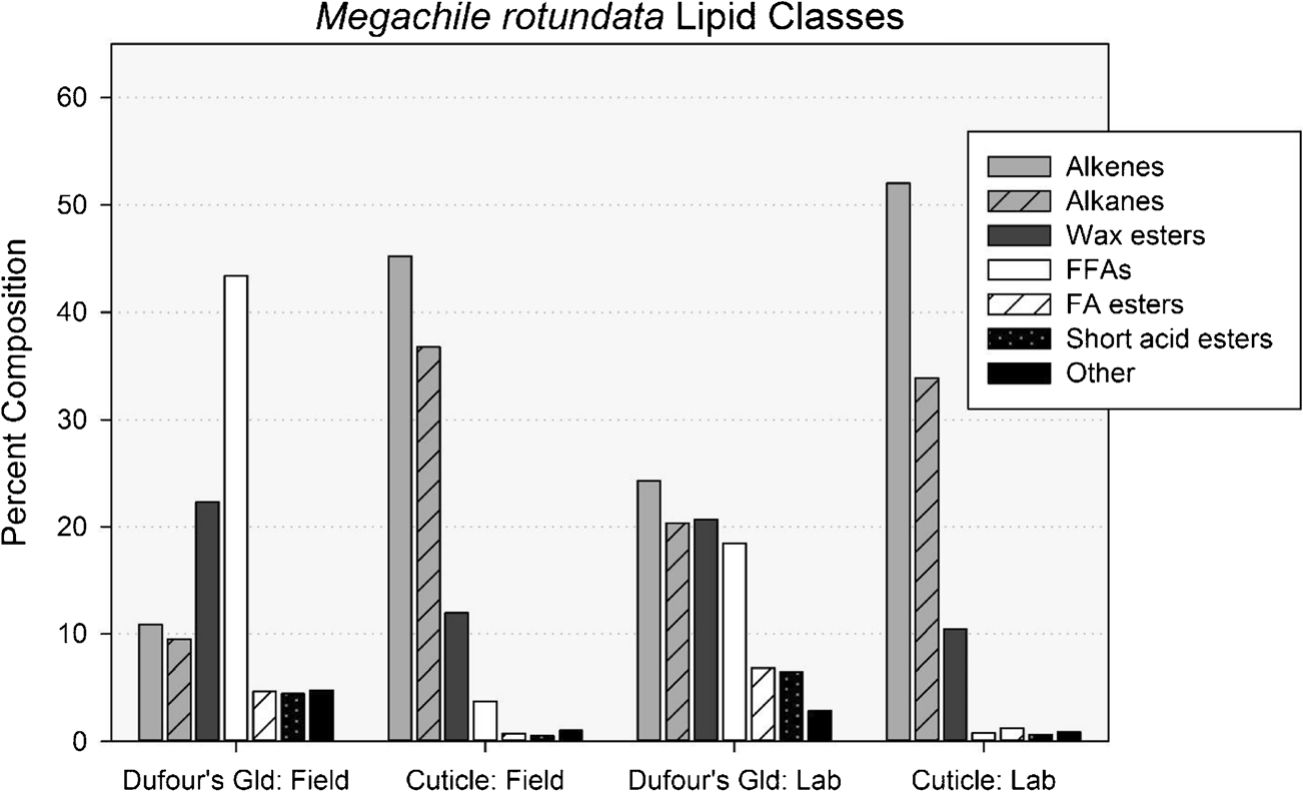
Percent composition of lipid classes found in solvent extracts of the cuticles and Dufour’s glands of field-collected and laboratory-reared *Megachile rotundata* females. *Alkenes* mono-alkenes, *Alkanes n*-alkanes, methyl-branched and oxo-alkanes, *Wax esters* esters of long-chain acids and long-chain alcohols, *FFAs* free fatty acids, *Short acid esters* esters of acetic or butyric acid and long-chain alcohols, *FA esters* esters of long-chain acids and ethyl or isopropyl alcohol, *Other* long-chain aldehydes, sterols and unknowns

Identification of *n*-alkanes, fatty acid ethyl and isopropyl esters, and free fatty acids were based on comparison of retention times and mass spectra of authentic standards (Restek Corp., Bellefonte, PA; Nu-Chek-Prep, Inc., Elysian, MN). Aldehydes (ALD) were identified based on comparison of their mass spectra with standards synthesized as described in Buckner et al. (1994). The mass spectra of all other classes of lipids were similarly interpreted: methyl-branched alkanes synthesized according to Coudron and Nelson (1978) and Nelson and Sukkestad (1970); alkenes according to Buckner et al. (2009); wax esters as well as butyrate and acetate esters of long-chain alcohols according to Buckner et al. (1994). Multiple positional isomers of C23 – C31 mono-alkenes were resolved into one to three different peaks (Figs. 1 and 2) that were quantified separately, and the total mass at each chain length was summed (Tables 1 and 2). Due to the extremely small mass of lipid obtained from extracting each individual bee cuticle and her Dufour’s gland and to the large number of different lipids identified, we did not attempt to quantify the various isomers separately.

**Table 2:** The average percentage composition (± SE) of compounds in lipid extracts of the cuticles and Dufour’s glands from field-collected (*N* = 10) and laboratory-reared (*N* = 11) *Osmia lignaria* females

Peak^b^	Compound	Mean percent composition^a^ ± SE			
		Cuticle		Dufour’s Gland	
		Field	Laboratory	Field	Laboratory
1	14:0 FFA^c^	0.4 ± 0.1	0.2 ± 0.1	0.9 ± 0.2	0.3 ± 0.1
2	16:1 FFA	1.2 ± 0.1	t	4.8 ± 0.8	1.1 ± 0.3
3	16:0 FFA	1.4 ± 0.2	0.3 ± 0.1	2.7 ± 0.3	0.7 ± 0.2
4	16:1 FAEE	–	0.3 ± 0.1	–	1.4 ± 0.6
5	16:0 FAEE	–	t	–	t
6	16:1 FAIPE	–	t	–	0.2 ± 0.1
7	18 ALD	–	t	–	–
8	16:0 FAIPE	–	t	t	t
9	21:0 HC	–	t	–	t
10	18:U^d^ FFA	5.5 ± 1.0	0.2 ± 0.1	24.5 ± 3.7	5.1 ± 2.0
11	Terpenoid #1	–	t	–	0.8 ± 0.1
12	18:0 FFA	1.1 ± 0.2	0.3 ± 0.1	2.9 ± 0.5	0.7 ± 0.2
13	18:1 FAEE	–	0.3 ± 0.1	t	1.2 ± 0.2
14	Terpenoid #2	–	t	–	1.6 ± 0.4
15	18:1 FAIPE	–	0.3 ± 0.1	0.1 ± 0.1	1.4 ± 0.3
16	22:0 HC	–	t	–	t
17	20 ALD	–	t	–	–
18	23:1 HC^e^	–	t	t	t
19	23:0 HC	0.3 ± 0.1	1.6 ± 0.1	0.3 ± 0.1	1.6 ± 0.1
20	20:1 FFA	–	t	–	0.7 ± 0.3
21	20:1 FAEE	–	–	–	t
22	20:0 FFA	–	t	–	t
23	24:1 HC	0.0 ± 0.0	t	0.2 ± 0.0	0.2 ± 0.1
24	20:1 FAIPE	–	t	–	t
25	24:0 HC	0.3 ± 0.0	0.4 ± 0.0	0.2 ± 0.0	0.3 ± 0.0
26	22 ALD	–	t	–	–
27	25:1 HC	12.4 ± 0.9	18.4 ± 0.8	13.8 ± 1.5	18.9 ± 1.9
28	25:0 HC	13.9 ± 0.7	18.1 ± 0.5	9.2 ± 1.6	14.5 ± 2.4
29	Terpenoid #3	–	t	–	t
30	25A^f^ HC	–	t	–	t
31	Unknown #1	–	t	–	t
32	26:1 HC	1.2 ± 0.0	1.3 ± 0.0	0.9 ± 0.1	1.1 ± 0.1
33	Unknown #2	–	–	t	t
34	26:0 HC	0.5 ± 0.0	0.3 ± 0.0	0.2 ± 0.0	0.2 ± 0.0
35	24 ALD	t	t	–	–
36	Terpenoid #4	–	t	–	t
37	27:1 HC	29.5 ± 1.3	29.2 ± 1.2	21.7 ± 1.7	24.7 ± 2.2
38	27:0 HC	5.9 ± 0.7	4.8 ± 0.2	3.2 ± 0.7	4.0 ± 0.8
39	Terpenoid #5	–	t	t	t
40	Unknown #3	–	t	–	t
41	27A HC	–	t	–	t
42	Unknown #4	–	t	–	t
43	28:1 HC	0.4 ± 0.0	0.5 ± 0.0	0.3 ± 0.0	0.3 ± 0.0
44	28:0 HC	0.2 ± 0.0	t	t	t
45	26 ALD	t	t	–	–
46	Unknown #5	–	t	–	–
47	29:1 HC	11.1 ± 0.5	9.3 ± 0.2	7.0 ± 0.7	7.8 ± 0.6
48	29:0 HC	1.9 ± 0.3	1.6 ± 0.1	0.9 ± 0.2	1.3 ± 0.3
49	Terpenoid #6	–	t	t	0.6 ± 0.3
50	29A HC	–	t	–	t
51	Terpenoid #7	–	–	–	t
52	Terpenoid #8	–	t	–	0.2 ± 0.1
53	Terpenoid #9	–	t	–	0.2 ± 0.1
54	30:1 HC	t	t	t	t
55	30:0 HC	t	t	0.1 ± 0.1	t
56	28 ALD	–	t	–	–
57	C27 sterol	–	t	t	t
58	31:1 HC	2.2 ± 0.1	1.4 ± 0.1	0.9 ± 0.1	1.0 ± 0.0
59	31:0 HC	1.3 ± 0.2	0.9 ± 0.1	0.5 ± 0.1	0.7 ± 0.2
60	Terpenoid #10	–	t	–	0.8 ± 0.4
61	Terpenoid #11	–	t	–	t
62	32:0 HC	–	t	t	–
63	C29 sterol	–	–	0.7 ± 0.2	t
64	33:0 HC	0.3 ± 0.1	t	t	t
65	33A HC	t	t	t	–
66	32:0 WE	–	t	–	–
67	Terpenoid #12	–	t	–	1.4 ± 0.4
68	Terpenoid #13	–	t	t	0.4 ± 0.1
69	35:0 HC	–	t	–	t
70	34:1 WE	–	t	–	t
71	Unknown #6	–	t	–	–
72	Terpenoid #14	–	t	t	0.9 ± 0.4
73	Terpenoid #15	–	t	t	t
74	36:1 WE	–	0.2 ± 0.0	–	t
75	36:0 WE	–	t	–	–
76	Terpenoid #16	–	t	t	1.2 ± 0.4
77	38:U^g^ WE	–	0.2 ± 0.0	–	–
78	38:0 WE	–	t	–	–
79	40:U WE	1.0 ± 0.0	0.5 ± 0.1	0.6 ± 0.3	0.2 ± 0.0
80	40:0 WE	t	0.1 ± 0.1	t	t
81	42:U WE	3.7 ± 0.2	2.9 ± 0.3	2.1 ± 0.7	0.6 ± 0.1
82	44:U WE	2.2 ± 0.1	1.8 ± 0.2	0.2 ± 0.0	0.2 ± 0.0
83	46:U WE	1.9 ± 0.2	1.4 ± 0.1	t	t
84	48:1 WE	–	0.4 ± 0.1	–	–
85	50:1 WE	–	t	–	–

^a^Percent composition calculated from the integrated peak area from the GC-FID response as described in Materials and Methods

^b^Peak numbers correspond to those in Fig. 2

^c^Lipid classes were abbreviated as follows: *FFA* free fatty acid, *FAEE* fatty acid ethyl ester, *FAIPE* fatty acid isopropyl ester, *ALD* aldehyde, *HC* hydrocarbon, *WE* wax ester. The numbers to the *left* and the *right* of the colon for hydrocarbons, free fatty acids, fatty acid esters and wax esters are the total number of carbons and the number of double bonds, respectively

^d^U, A mixture of free fatty acids was identified with one, two and/or three points of unsaturation at this chain length

^e^Multiple positional isomers of C23 – C31 mono-alkenes resolve into one to three different peaks in Fig. 2, and the total quantity at each chain length is summed on one line in this table

^f^A, The hydrocarbon of indicated chain length has a single methyl branch internal to the molecule

^g^U, A mixture of wax esters was identified with one or one and two points of unsaturation in some samples at this chain length

### Principal Component/Linear Discriminant Analysis for Cuticles and Dufour’s Glands

To reduce the complexity of the comparisons between the cuticular and glandular lipids of laboratory-reared and field-collected *M. rotundata* and *O. lignaria* populations (hereafter referred to as “laboratory” and “field” bees), Principal Component Analysis (PCA) was performed, and the principal component (PC) values were then used for Linear Discriminant Analysis (LDA). The multidimensional analyses portrayed in two dimensions showed groupings of the lipid profiles of cuticles and Dufour’s glands according to whether they came from laboratory or field populations. Individual female lipid profiles (percent composition of each compound) from the cuticle and Dufour’s gland were obtained by dividing the mass of each unique lipid by the total mass of lipids from each bee, resulting in a proportion value. To perform PCA and LDA of the lipid profiles, we used the “prcomp” function in R version 3.1.1 (R Core Team 2016). LDA was performed using the ‘lda’ function from the package MASS (Venables and Ripley 2002).

PCA identified 46 PCs for *M. rotundata* and 42 PCs for *O. lignaria*. Factor reduction was based visually on the screenplots and by comparisons of LDA plots using three to many PCs until no further clarity in groupings was achieved. We subsequently chose to use the first three and six PCs for *M. rotundata* and *O. lignaria*, respectively (Supplementary Figs. 1 & 2). To implement the ‘lda’ function, all data from cuticles and glands and for the bee environment (laboratory or field) were collapsed to a single factorial level. Then for each species, an LDA model was fitted in which this factor was the response of the PCs. Once linear discriminant functions were calculated, we visually examined differences among lipid sources (cuticles and glands) by environment by plotting the calculated scaling values for each individual’s profiles. Although individuals were collected across different years for each species, sample types did not group only according to years.

### Correlative Matching of Cuticles and Dufour’s Glands within Individuals

To determine if lipid composition of the cuticle and Dufour’s gland of the same female were uniquely matched, we investigated whether one or more components of the cuticle were good predictors of the composition of the Dufour’s gland of the same extracted bee (and vice-a-versa). We used Pearson Correlations (SAS 2002-2012, SAS Institute Inc., Cary, NC, USA) for relating the percent composition of each component of cuticular and glandular profiles of bees from the laboratory and the field separately. Because too many zero values negated meaningful results for the correlations, any given compound having zeroes for all glandular or cuticular samples was removed from the data prior to analysis. We report only the results for compounds showing Pearson Correlation values greater than 0.60, *P* < 0.03, although other compounds were significant at *P* < 0.05.

## Results

### Chemistry of *Megachile rotundata*

Ninety compounds were quantified from solvent extracts of the cuticles and the Dufour’s glands of *M. rotundata* females (Table 1, Figs. 1 and 2). The major lipid classes present in *M. rotundata* female cuticles of both laboratory and field bees (92% and 96%, respectively) were mono-alkenes, *n*-alkanes, and WEs (Figs. 1a and 2). The same three lipid classes were the most prominent components of extracts of Dufour’s glands of the laboratory females (65%) (Fig. 1b), but FFAs dominated the composition of the glandular extracts of field bees (43%) (Fig. 2). The concentration of hydrocarbons (alkenes and alkanes) was higher on the cuticles than in the Dufour’s glands, and cuticular extracts contained small percentages of methyl-branched alkanes and aldehydes not seen in Dufour’s gland extracts. WEs, acetate and butyrate esters of fatty alcohols, and fatty acid ethyl and isopropyl esters were also detected and represented a larger proportion of lipids in the Dufour’s glands than of lipids on the cuticles. All sample types also contained sterols, oxo-alkanes, and two frequently-detected unknown compounds, i.e., peaks with mass spectra that resemble insect lipids but could not be identified (Table 1, Figs. 1 and 2).

The main compounds in cuticular extracts of laboratory females were pentacosenes, pentacosane, heptacosenes, heptacosane and nonacosane, in order of abundance (Table 1). The same five abundant compounds were present in the cuticular extracts of field bees, except having a higher percentage of heptacosenes than pentacosane. The most prevalent compounds (39.4%) in lipid profiles of Dufour’s glands of laboratory bees were pentacosenes, pentacosane, hexadecanoic and octadecenoic acids, whereas octadecenoic, hexadecanoic and octadecanoic acids, and pentacosenes (in descending order) were predominant (46.5%) in glandular profiles of field bees (Table 1).

The percentage of wax esters in Dufour’s gland extracts was double that in cuticular extracts, but was limited to fewer isomers (Table 1). Cuticular extracts included 16 saturated (with 18–48 carbons) and 12 unsaturated (with 26–48 carbons) even carbon number WEs, while the Dufour’s gland extracts included 12 saturated (with 18–40 carbons) and eight unsaturated (with 26–40 carbons) WEs. The 46-carbon unsaturated WE had the highest percent composition in 21 of 24 cuticular extracts, whereas eight different WEs comprised the highest percent composition for the 22 individual Dufour’s gland samples, ranging from 26 to 34 carbons.

The percentage of FFAs was nearly 3.8% of the cuticular extracts of field bees, almost 5-fold higher than the cuticular composition (0.8%) of laboratory bees. Additionally, the distribution and relative compositions of the acids with even numbers of carbons, including saturated acids with 14–20 carbons and unsaturated acids with 18 and 20 carbons, closely resembled the distribution and composition of FFAs seen in lipid profiles of previously analyzed solvent extracts of alfalfa leaves and pollen (Buckner and Hagen, unpublished results). In an earlier study (Guédot et al. 2013), we had treated these FFAs as contaminants and disregarded them in compositional calculations for bee lipid profiles. This current study contradicts our former assumption with the finding that Dufour’s glands of field bees contained 43.4% FFAs, more than the next three “major” lipid classes combined. Additionally, the Dufour’s gland extracts of laboratory bees, which were never exposed to alfalfa, contained a large percentage of FFAs (18.5%).

Field females weighed roughly 12% less than laboratory females, and the average total mass of lipid extracted from their cuticles and glands was about 50% and 73%, respectively, of the amount of lipid extracted from laboratory bees. When the mass of lipid extracted from each bee’s Dufour’s gland was compared to the amount found on the same bee’s cuticle, a slightly higher percentage, 2.9%, was obtained from field bees compared to 2.0% from laboratory bees.

### Chemistry of *Osmia lignaria*

**Fig. 3:**
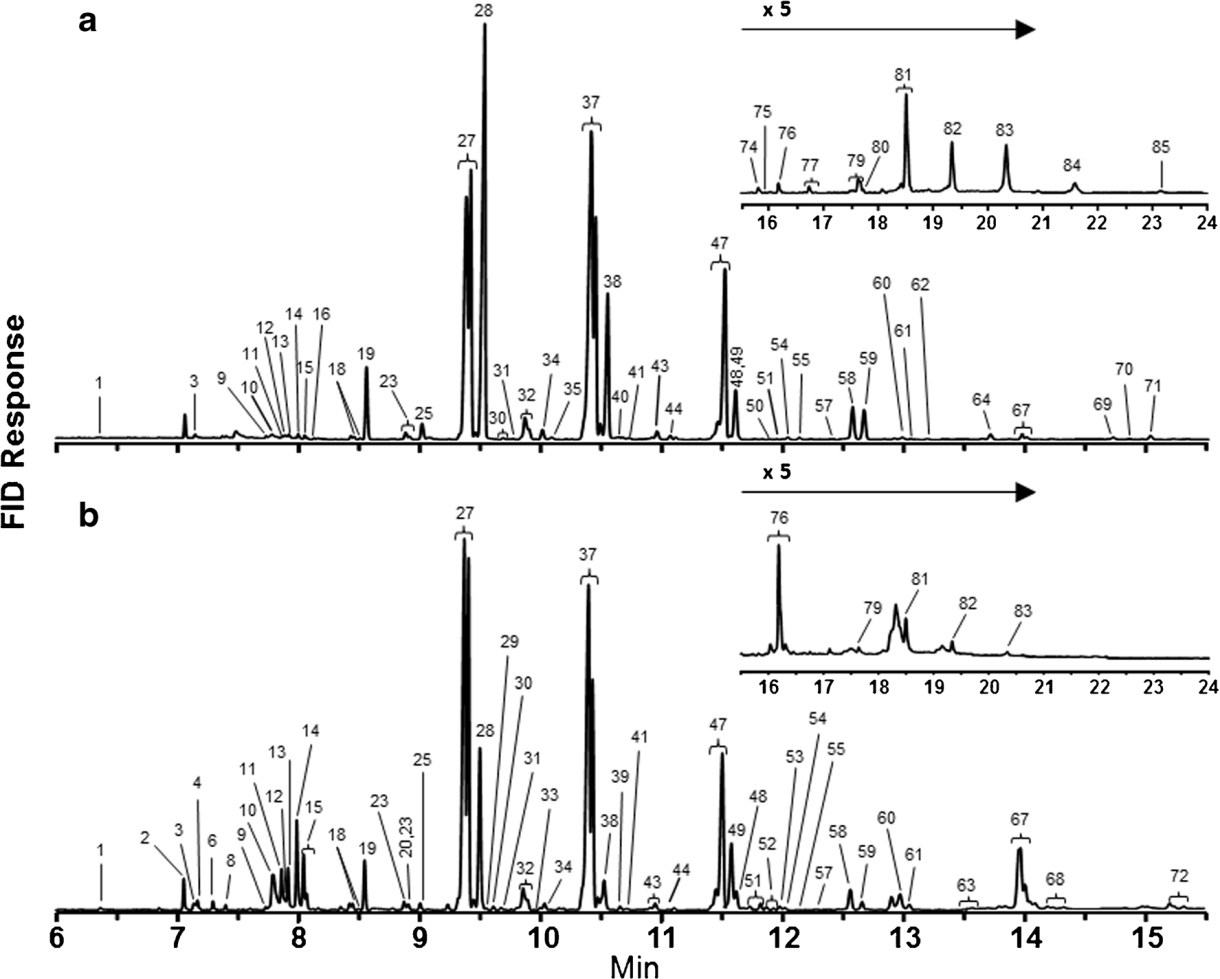
Gas chromatography (GC) flame ionization detector (FID) results of analyses of extracts from the (**a**) cuticle and (**b**) Dufour’s gland of one representative *Osmia lignaria* female. Identifications of known, numbered peaks are reported in Table 2. Noticeable GC peaks that are unlabeled were of insufficient concentration to create a peak in the GC-MS chromatogram

**Fig. 4:**
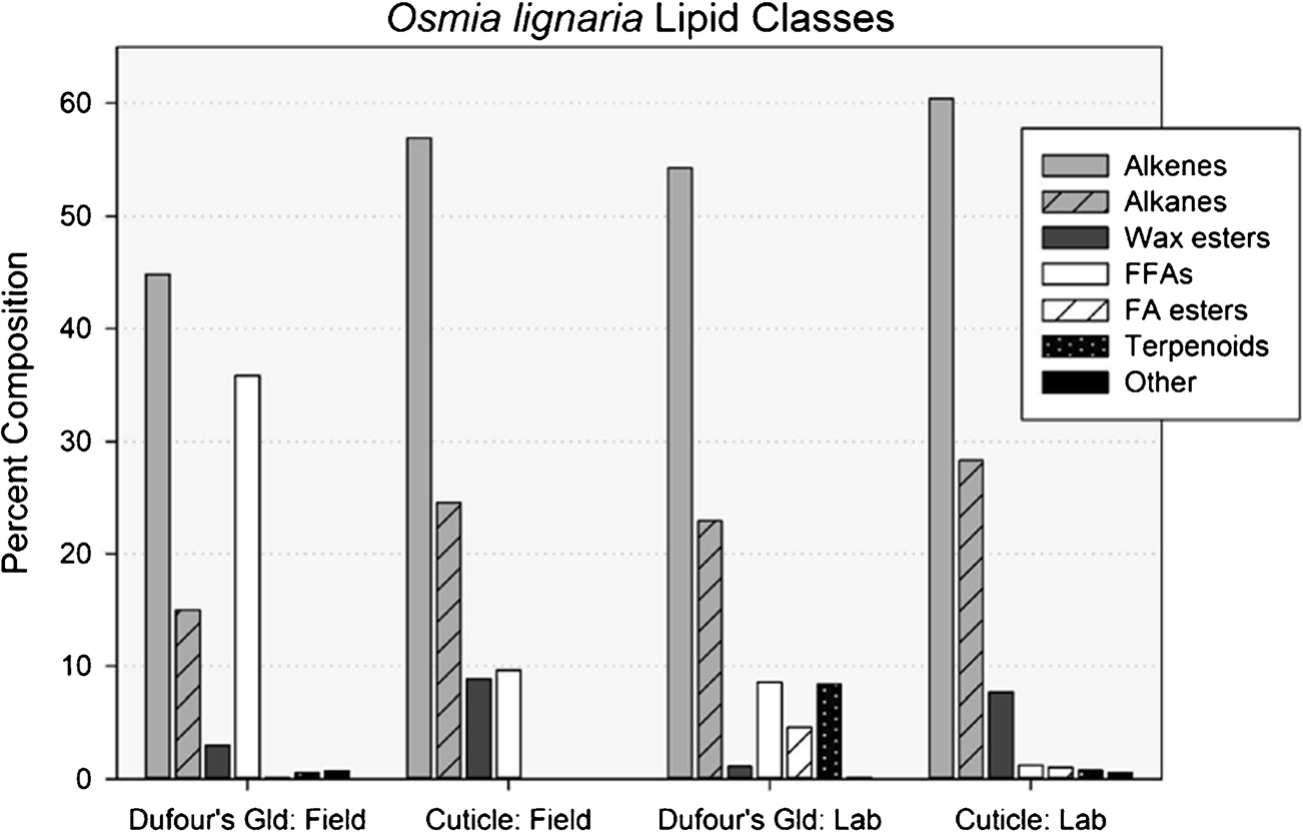
Percent composition of lipid classes found in solvent extracts of the cuticles and Dufour’s glands of field-collected and laboratory-reared *Osmia lignaria* females. *Alkenes* mono-alkenes, *Alkanes n*- and methyl-branched alkanes, *Wax esters* esters of long-chain acids and long-chain alcohols, *FFAs* free fatty acids, *FA esters* esters of long-chain acids of ethyl or isopropyl alcohol, *Terpenoids* probable identifications, *Other* long-chain aldehydes, sterols and unknowns

Eighty-five compounds were quantified from the solvent extracts of cuticles and Dufour’s glands of *O. lignaria* females (Table 2; Figs. 3 and 4). In order of abundance, the major lipid classes present were mono-alkenes, *n*-alkanes, and WEs. These compounds comprised over 90% and 96% of field and laboratory cuticular lipids, respectively. Cuticular extracts also contained FFAs (higher amounts in field bees than in laboratory bees), methyl-branched alkanes, and aldehydes (Table 2; Figs. 3a and 4). Small quantities of terpenoids, sterols, several unknowns as well as fatty acid ethyl (FAEE) and isopropyl esters (FAIPE) were also present in laboratory bee cuticular extracts and laboratory and field bee Dufour’s glandular extracts, but not in cuticular extracts of field bees. Mono-alkenes, *n-*alkanes, and FFAs were most prominent in Dufour’s gland extracts, with higher percentages of FFAs and WEs in field bees than in laboratory bees (Table 2; Figs. 3b, 4). Aldehydes were not present in the Dufour’s gland extracts.

For all four *O. lignaria* sample types, the mixtures of mono-alkenes, *n-*alkanes and very small quantities of mono-methyl-branched alkanes were mainly odd-numbered carbon chains ranging from 23 to 31 (Table 2). For cuticular lipids of both field and laboratory females, over 29% of the total lipid was comprised of heptacosenes. Other major cuticular components included pentacosane, pentacosenes, nonacosenes and heptacosane (Table 2). The major components of the Dufour’s gland were identical to the cuticular lipids with one notable difference, the inclusion of octadecenoic acid (18:1 FFA) as the most prevalent compound in the Dufour’s glands of field bees and the fifth most of laboratory bees.

For *O. lignaria* cuticular and glandular lipid profiles for both the laboratory and field bees, the FFAs as well as the FAEE and FAIPE were comprised of even-numbered saturated (14–20 carbons) and unsaturated (16:1, 18:1, 18:2 and 18:3) acids (Table 2). However, 18:3 and 18:2 FFAs were present in larger and highly variable amounts only in the cuticles of field-reared bees. Oleic acid (18:1) was the primary 18-carbon unsaturated acid in all samples types. In field-reared cuticle samples, large quantities of 18:3 and lesser amounts of 16:0 and 18:2 FFAs were present in some samples similar to our current understanding of FFA profiles of *O. lignaria* pollen extracts (contradicts Guédot et al. 2006) that are 56% 18:3, 32% 16:0, 9% 18:2 and 3% 18:0 with no 18:1 nor 16:1 FFAs present. In Dufour’s gland samples of both laboratory- and field-reared bees, the second most prevalent acid was hexadecenoic acid (16:1 FFA) not found in *P. tanacetifolia* pollen. FFA profiles of nesting tubes (Guédot et al. 2006) more closely resembled the FFA profile of pollen, with large but variable amounts of 18:3 followed by 16:0 or 18:0, small amounts of 18:2 FFAs, only trace amounts of 18:1 FFA, and no 16:1 FFA (unpublished results). Similar to *M. rotundata*, FFAs comprised a disproportionately high percentage of the lipid extracted from the glands of field bees exposed to *P. tanacetifolia* and other components of the nesting and foraging environment (35.8%) compared to the laboratory bees that were never exposed (8.6%) (Table 2, Fig. 2). However, selected ion monitoring (SIM) analyses of GC-MS data from cuticular extracts of laboratory-reared bees showed the presence of both 16- and 18-carbon mono-unsaturated FFAs that are not present in *P. tanacetifolia* pollen.

Sixteen different compounds were quantified and labelled as terpenoids in *O. lignaria* samples. The mass spectrum of each was characterized by a base peak of 69 and the same ion intensity pattern formed by ions 69, 81, 93, 107, 121 and 136. While the mass spectra of several did match well with geranyl and farnesyl fatty acid esters (Tengö and Bergström 1975), no further work was done to identify these compounds conclusively, which accounted for less than 2% of lipid for any individual sample except the laboratory Dufour’s gland extracts (2–16%) (Supplementary Tables 1 and 2).

On average, laboratory *O. lignaria* had >1.5 times more cuticular and glandular lipids than field bees. The amount of lipid extracted from the cuticles varied less between samples than between the amount of lipid in individual Dufour’s gland samples (Table 3). Although field and laboratory bees were approximately the same age when freeze killed (~3 days old), their nesting experience, body mass, and food source differed. Nonetheless, in each sampling year, solvent extracts of the cuticle yielded between 12 to 20 times more lipid than that extracted from the same female’s Dufour’s gland (Table 3).

**Table 3:** The number of samples, age of bees when sampled, mean lipid mass (± SE) (μg), and lipid mass range per female for cuticle and Dufour’s gland of extracted lipids from the cuticles and Dufour’s glands of field-collected and laboratory-reared *Megachile rotundata* and *Osmia lignaria* females

Source	*Megachile rotundata*		*Osmia lignaria*	
	Field	Laboratory	Field	Laboratory
N; age	10; 2–4 wks	12; 3–7 days	10; 2–7 days	11; 2–3 days
Cuticle Mean	31.02 ± 2.13	60.77 ± 4.21	74.02 ± 8.09	127.96 ± 7.73
Cuticle Range	21.94–40.51	37.16–89.49	30.63–110.32	92.07–166.73
Dufour’s Gland Mean	0.81 ± 0.21	1.09 ± 0.26	7.72 ± 2.70	13.01 ± 2.91
Dufour’s Gland Range	0.41–2.61	0.28–3.35	2.73–31.66	3.71–33.24

### Linear Discriminant Analysis of Principal Component Loadings for Lipid Profiles

**Fig. 5:**
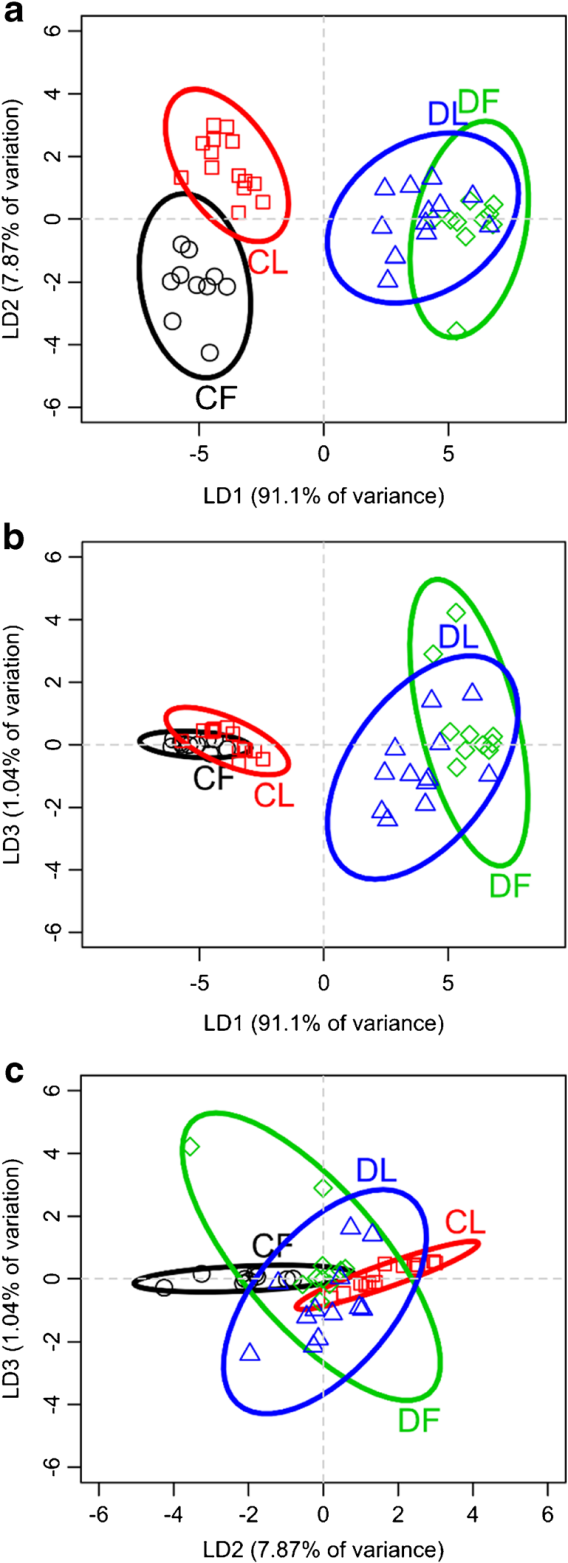
Results of linear discriminant analysis (LDA) of the first three principal components from a Principal Components Analysis of lipid composition from cuticle (C) and Dufour’s glands (D) from adult *Megachile rotundata* that were collected in either field (F) or laboratory (L) conditions. **a**, **b** Linear Discriminant 1 (LD1) generated strong contrasts between the cuticles and Dufour’s glands, regardless of whether they were collected from the field or laboratory. **a**, **c** LD2 showed a distinction between the cuticle lipid profiles of field vs laboratory bees. **b**, **c** LD3 showed a very little contrast between the lipid profiles of bees from the laboratory and the field

Clear distinctions were apparent between lipid profiles of *M. rotundata* cuticles and Dufour’s glands and between cuticles (but not glands) of laboratory and field bees (Fig. 5). Linear Discriminant 1 (LD1) accounted for 91% of the variation among lipid profiles and showed the strong differences between the cuticles and Dufour’s glands (Fig. 5a, b). LD2 accounted for 8% of the variance in lipid profiles (Fig. 5a, c), revealing a distinct difference between the laboratory and field bee cuticles. LD3 accounted for only 1.0% of the variation, showing only slight separation of the Dufour’s gland profiles according to environment, but no difference between groups of cuticular profiles (Fig. 5b, c).

**Fig. 6:**
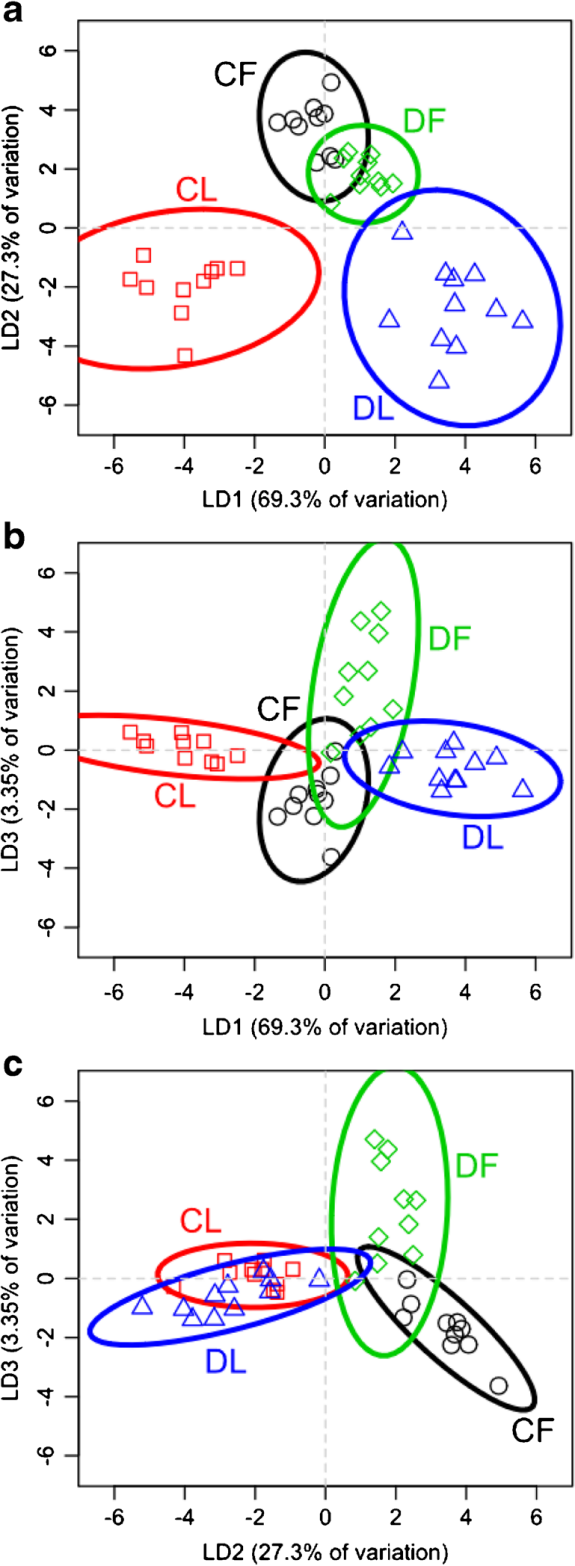
Results of linear discriminant analysis (LDA) of the first three principal components from a Principal Components Analysis of lipid composition from cuticle (C) and Dufour’s glands (D) from adult *Osmia lignaria* that were collected in either field (F) or laboratory (L) conditions. **a**, **b** Linear Discriminant 1 (LD1) showed clear contrasts among both cuticles and Dufour’s glands and whether bees were collected from the field or laboratory. **a**, **c** LD2 showed a distinction between laboratory and field bees. **b**, **c** LD3 showed some contrast between the lipid profiles of cuticles and glands of field bees

Compared to the results for *M. rotundata*, LDA for *O. lignaria* samples revealed distinct differences between the lipid profiles of cuticles and Dufour’s glands in the laboratory bees, but overlap in the profiles of the cuticles and glands of field bees (Fig. 6). LD1 accounted for 69% of the variation, which revealed differences between the laboratory bee profiles (Fig. 6a, b). LD2 accounted for 27% of the variation, showing clear contrasts between the cuticular and glandular lipid profiles (Fig. 6a, c). LD3 accounted for 3% of the variation in lipid profiles, clearly distinguishing between the cuticular and glandular profiles for field bees, and less distinction for the laboratory bees (Fig. 6b, c).

### Pearson Correlation Analyses

**Fig. 7:**
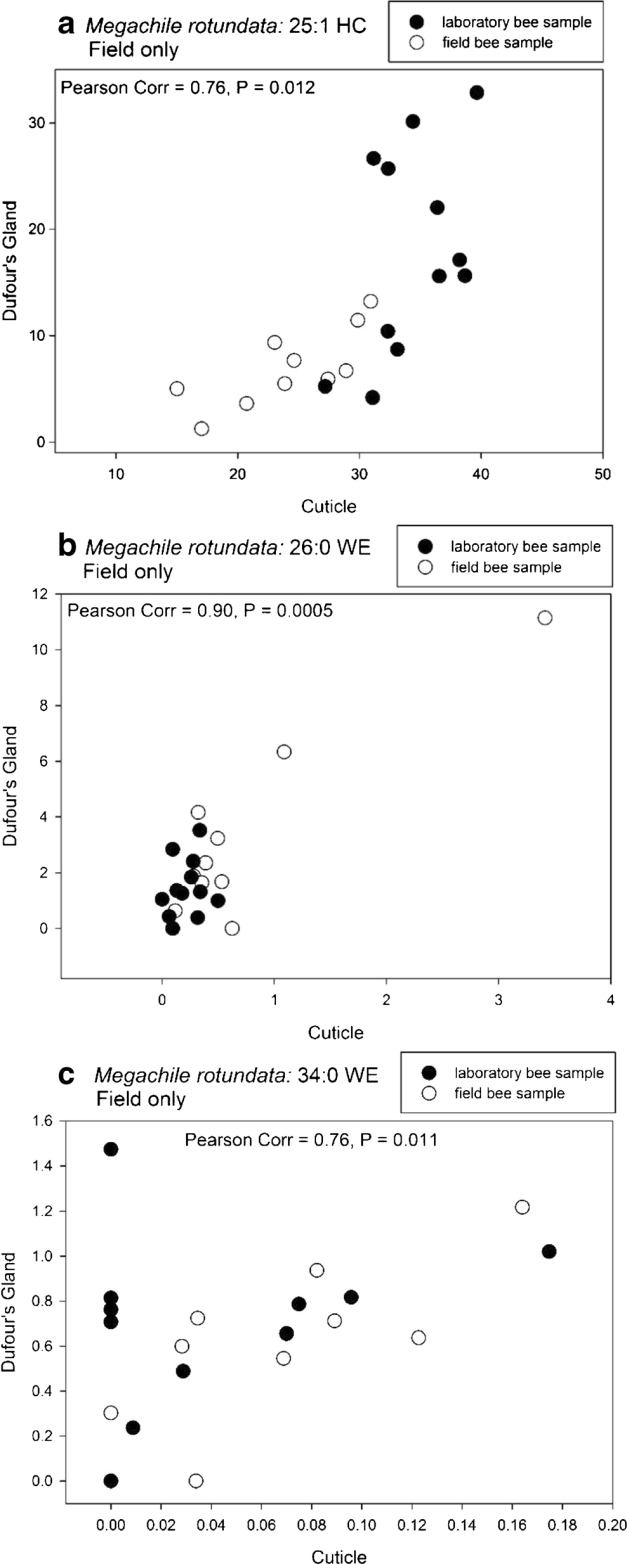
Pearson correlation for the percent composition of (**a**) the 25-carbon alkene (25:1 HC), (**b**) the 26-carbon saturated wax ester (26:0 WE), and (**c**) the 34-carbon saturated wax ester (34:0 WE) from the matching profiles of *Megachile rotundata* cuticles and Dufour’s glands for bees taken in laboratory and field conditions

For *M. rotundata*, the percent compositions of most of the compounds common to both the cuticle and Dufour’s gland were not highly correlated (i.e., were <65% correlated), although several were significant at *P* < 0.05. The relative proportions of 25:1 HC (Fig. 7a), 26:0 WE (Fig. 7b), and 34:0 WE (Fig. 7c) were found to be most strongly and positively correlated between the cuticle and the Dufour’s gland of the same female for only the field bees. No compound was found to be highly correlated between the cuticular and glandular lipids of the laboratory females.

**Fig. 8:**
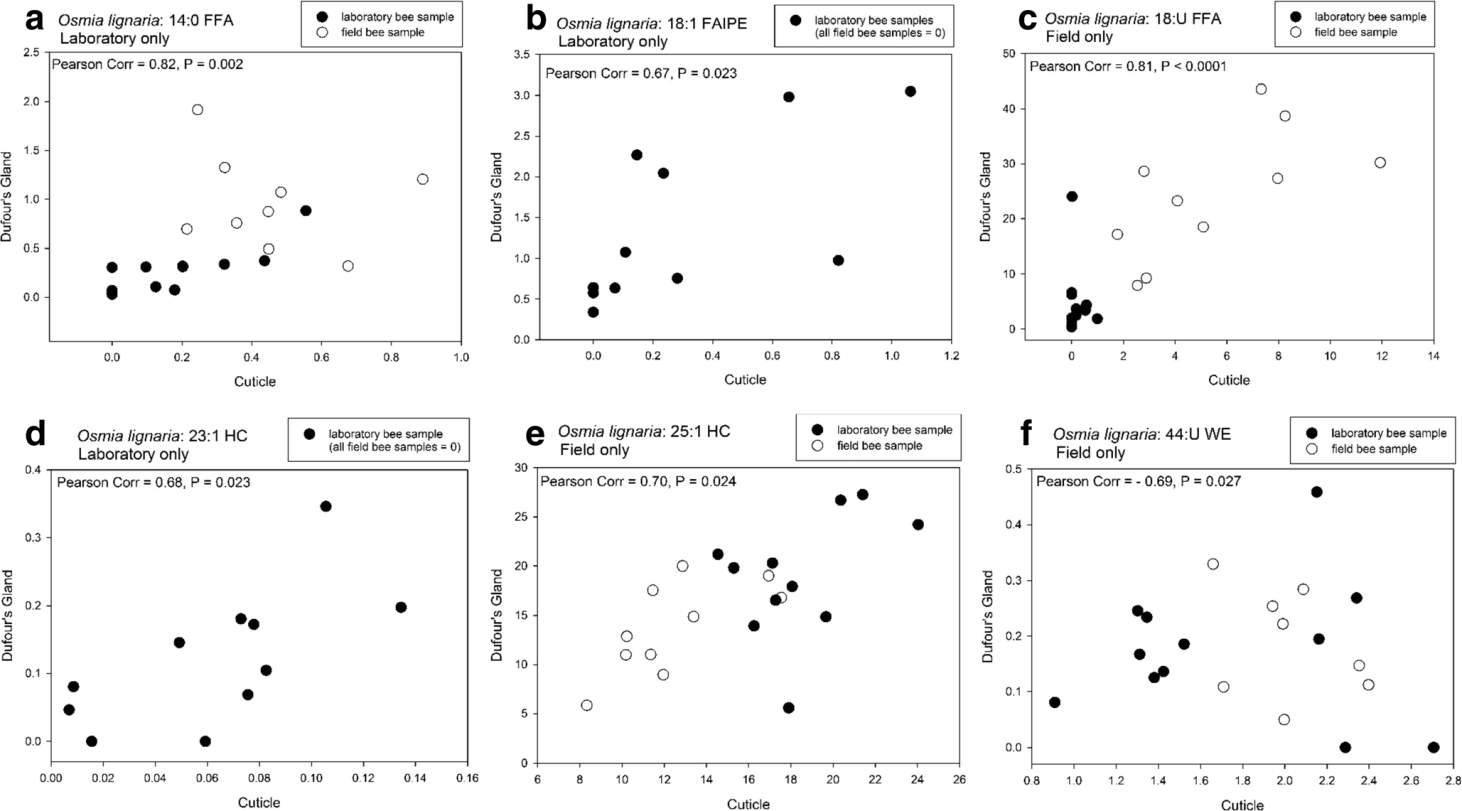
Pearson correlation for the percent composition of (**a**) the 14-carbon, saturated free fatty acid (14:0 FFA), (**b**) the 18-carbon fatty acid isopropyl ester (18:1 IPE), (**c**) the 18-carbon unsaturated free fatty acid (18:U FFA), (**d**) the 23-carbon alkene (23:1 HC), (**e**) the 25-carbon alkene (25:1 HC), and (**f**) the 44-carbon unsaturated wax ester (44:U WE) from the matching profiles of *Osmia lignaria* cuticles and Dufour’s glands for bees taken in laboratory and field conditions

For *O. lignaria*, Pearson Correlation analysis revealed relative amounts of several compounds common to both the cuticle and Dufour’s gland to be significantly correlated, but not always for bees from each collection environment (Fig. 8). Strong positive correlations for laboratory bee data were revealed for the proportions of 14:0 FFA (Fig. 8a) and 18:1 FAIPE (Fig. 8b). For field bee data, positive strong correlations were found for 18:U FFA (Fig. 8c), 23:1 HC (Fig. 8d), and 25:1 HC (Fig. 8e). A strong negative correlation was also found in field bee data for the 44:U WE (Fig. 8f).

## Discussion

The secretion of long-chain hydrocarbons is common to all hymenoptera whose Dufour’s chemistry has been analyzed (Mitra 2013). Adding to the modest number of reports of solitary bee lipid chemistry (Mitra 2013), this is the first examination of the lipid composition of the Dufour’s glands of cavity-nesting *M. rotundata* and *O. lignaria*. We found the lipid profiles of cuticles and Dufour’s glands of these species to have distinct compositions. The profiles from bees maintained in the laboratory and those actively nesting and exposed to the environment were clearly different in *M. rotundata*, but not as distinguishable in *O. lignaria*. Our findings of cuticular chemistry concur with previous studies for *M. rotundata* and *O. lignaria* (Buckner et al. 2009; Guédot et al. 2006, 2013), showing that the major lipid classes of the cuticle are alkanes and alkenes. The newly examined Dufour’s gland profiles also are composed mainly of hydrocarbons, in addition to abundant WEs and FFAs, with the latter being especially abundant in the glands of nesting bees.

For this study, we were able to examine adult bees of different ages, with and without nesting experience, but all having had the chance to mate. Due to their developmental histories, age at emergence from the cocoon after winter diapause differs between *M. rotundata* and *O. lignaria*. Wintering cocoons of *M. rotundata* contain diapausing prepupae (larvae in the ultimate, fifth instar that have spun a cocoon), while *O. lignaria* cocoons contain diapausing adults. Therefore, the three- to seven-day old, laboratory-reared *M. rotundata* females in this study became adults just prior to chewing out of their cocoons in the summer, but the *O. lignaria* females had been adults for eight to nine months (having eclosed in late summer/early fall) before chewing out of cocoons in the spring. The deposition of lipids onto the outer epicuticle occurs just before ecdysis (Klowden 2007), and lipid deposition may continue or may change with ecological and physiological conditions (Chung and Carroll 2015). The lipid profiles of the *O. lignaria* adults might, therefore, be considered more complete in their composition or complexity compared to profiles of *M. rotundata* adults. However, just as for *M. rotundata* females, there were noticeable differences between profiles of the non-nesting and nesting *O. lignaria* females, even though all of the *O. lignaria* sampled had been adults for a long time. Examination of newly-eclosed *O. lignaria* (unemerged) adults excised from cocoons in the fall may reveal a different lipid profile compared to the profiles of cuticles and Dufour’s glands of the spring-emerged bees studied thus far.

The cuticular extracts of three- to seven-day old laboratory *M. rotundata* females in our study contained the same predominant lipids (alkanes and alkenes) as those previously observed in just-emerged bees (Buckner et al. 2009). Despite the identical extraction and analysis procedures used in this study and the former, nearly three times as many different lipid components were present in extracts of our laboratory *M. rotundata* females, which were no more than one week older than the 24 h old bees. Although the lipid amounts also appeared to initially increase with maturation of the laboratory bees, the amount of cuticular lipids from the weeks-old field bees was less than that from laboratory bees. Cuticular lipid loss may occur if lipids are rubbed off of bees while active inside nests and if surface lipids are not replaced as bees grow old, or such lack of cuticular lipids may reveal that new lipid production is allocated for nest-marking rather than surface protection. Moreover, the older field bees presented a more diverse set of lipid compounds than younger laboratory bees. Most notable was the diversity of WEs on cuticles of older *M. rotundata* females, with 28 combined saturated and unsaturated compounds (12% of total lipids) compared to only seven different WEs (7% of total lipids) in the 12–24 h old, unmated bees (Buckner et al. 2009). The fact that female bees are in intimate contact with lipids found on walls of narrow tunnels during marking and provisioning of nests and while resting in or guarding tunnels (Guédot et al. 2013) may explain the differences between cuticular WEs of older nesting bees and non-nesting bees due to possible bidirectional transfer of lipids between tunnel walls and cuticles.

In contrast to the cuticles, the Dufour’s gland of laboratory and field *M. rotundata* females contained more than twice the percentage of WEs (over 20%). The majority of the WE chain lengths were 38 carbons or less in the Dufour’s glands, but cuticles had higher percentages of 40–48-carbon WEs. Indeed, lipid extracts of both the outer and middle *M. rotundata*-marked nesting tubes (Guédot et al. 2013) also each comprised approx. 17% WEs, which contained higher percentiles of these same WEs (≤38 carbons) than did the cuticular extracts of bees nesting in those tubes. Another similarity between the *M. rotundata* Dufour’s gland and nesting tube extracts is that no single WE chain length was consistently the most abundant compound in all samples (unlike the cuticle). Instead, the 22 Dufour’s gland samples in this study had eight different WE chain lengths at the highest relative amounts. Similar variation was found among the 12 outer nesting tubes, with the occurrence of seven different WE chain lengths at the highest concentration. GC-MS SIM analysis detected many different acid:alcohol chain length isomers at each WE chain length, and we propose that these compounds possess enough complexity to serve as individual nest recognition cues. Acetate esters were also roughly nine times more concentrated in the *M. rotundata* Dufour’s glands than the cuticles. The lipid profiles from the *M. rotundata* nesting tubes contained higher percentiles of these same compounds than did the cuticular extracts of bees nesting in those tubes (Guédot et al. 2013). Therefore, the presence of higher concentrations of acetate esters in nesting tubes is also consistent with the deposition of lipids from the Dufour’s gland during nest-marking.

There were only subtle differences between the cuticular and Dufour’s gland lipid compositions of *O. lignaria* females. Except for trace amounts of aldehydes in the cuticle, all lipid classes were detected in both cuticular and Dufour’s gland extracts. Additionally, there were no specific lipids produced in higher concentrations by the Dufour’s gland, such as was found for certain chain lengths of *M. rotundata* WEs. However, as noted in Guédot et al. (2006), the relative proportions of 25:1 HC to 25:0 HC and 27:1 HC to 27:0 HC found in cuticular extracts were half those found in either nesting tubes or the Dufour’s gland. This alkene:alkane ratio suggests the contribution of Dufour’s gland in *O. lignaria* nest-marking, but it does not exclude the contribution of the cuticle as well.

Although the relative higher abundance of FFAs in the Dufour’s glands of *M. rotundata* also distinguished glandular from cuticular profiles, the FFAs found on *M. rotundata* and *O. lignaria* cuticles and from the glass nesting tubes in the nest recognition studies were considered contaminants that originated from alfalfa pollen and leaf pieces (*M. rotundata*) or from *P. tanacetifolia* pollen (*O. lignaria*) (Guédot et al. 2006, 2013). These compounds were not quantified and instead were eliminated from calculations of the lipid percent composition. Thus, we cannot include the abundance of FFAs as evidence of the Dufour’s gland as the source of the recognition cue.

Nonetheless, just as in *M. rotundata* and *O. lignaria*, differences also have been found between the cuticular and the Dufour’s gland lipid profiles of the solitary sweat bee *Lasioglossum malachurum* (Hymenoptera: Halictidae), with the Dufour’s glands having relatively low abundances of alkanes and alkenes and more abundant concentrations of lactones (Soro et al. 2011). *Lasioglossum malachurum* nestmates use the Dufour’s gland secretions to mark the ground-nest entrance and form a gestalt colony odor used for nest recognition (Hefetz 1998; Soro et al. 2011). The Dufour’s glands of ground-nesting *M. integra* and *M. mendica* were found to contain short chain FFAs and triglycerides (comprised of fatty acids with 10 or less carbons) (Williams et al. 1986), and glands of ground-nesting *M. fortis*, *M. parallela*, *M. gemula*, and *M. xylocopoides* contained triglycerides with fatty acids in the range of 1–16 carbons (Cane and Carlson 1984). Our methodology for chemical analysis to examine lipids of two cavity-nesting megachilids did not detect any of the same lactones, FFAs or triglycerides that were found in the other solitary bees studied, but did detect an abundance of other compounds of longer chain-lengths and different classes. Future examinations of other cavity-nesting bees also may reveal interesting components and discover their functions in adult communication or brood protection.

For our study, the lipid profiles of cuticles and Dufour’s glands of both *M. rotundata* and *O. lignaria* were clearly differentiated with discriminant analyses, especially for *O. lignaria*. Predictability of grouping sample profiles by source and environment within each species was accurate, except for the Dufour’s gland profiles of *M. rotundata*. Most of the variation in *M. rotundata* profiles was between the cuticle and Dufour’s glands, while most of the variation in *O. lignaria* profiles was between laboratory and field bees. Life experiences differed between laboratory and field bees. Laboratory bees were young, never foraged, and were not building nests or laying eggs. The field *M. rotundata* had been flying in an alfalfa field for several weeks and nesting in commercial bee boards (Pitts-Singer 2008). Field *O. lignaria* were approximately the same age as the laboratory bees, but for their first few days of adult activity had been foraging among floral resources and nesting in provided wooden cavities. Therefore, lipid compositional differences were likely influenced by age, experience, physiological condition, food sources, and exposure to environmental conditions.

Increased amounts and/or changes in composition of lipids as insects age or experience a change in reproductive status has been similarly reported in the cuticles of paper wasps (Panek et al. 2001) and in the Dufour’s glands of *L. malachurum* (Ayasse et al. 1990), bumble bees (Abdalla et al. 1999; Amsalem et al. 2009a, b), stingless bees (Grajales-Conesa et al. 2007), and honey bees (Dor et al. 2005; Urbanová et al. 2008). For example, long-chain esters are found to signal fertility in honey bee queen Dufour’s glands and in workers with developed ovaries (Dor et al. 2005; Katzav-Gozansky et al. 2002).

Lastly, by examining both the cuticular and glandular lipids of *M. rotundata* and *O. lignaria*, we sought to identify specific lipid components that allow for unique signaling among the female bees. We expected that compositional similarity between a female’s cuticular and Dufour’s gland profiles, or certain components of them, would reveal these distinguishing components. Bees rely on unique signals to recognize their own nests (Guédot et al. 2006, 2013), and also potentially recognize their kin or nests of kin whose lipid profiles may be similarly composed. Recognition ability is, therefore, especially important for reproductively active female bees. Although many compounds were shared between cuticles and glands, only a few had high correlation values. Indeed, we found the strongest cuticular and glandular profile correlations for bees collected in the field. Some of these compounds were in the lipid classes found in higher relative abundances in the Dufour’s glands than in the cuticles, which specifically were 25:1 HC for both species, 26:0 and 34:0 WEs for *M. rotundata*, and 18:U FFA, 23:1 HC, and 44:U WE for *O. lignaria*. Two compounds - 14:0 FFA and 18:1 FAIPE - were correlated for only the laboratory *O. lignaria* females.

Our study has shown that lipids of the cuticle and Dufour’s glands are important in the nest recognition system of the *M. rotundata* and *O. lignaria*. We found evidence that the Dufour’s gland plays a major role as the source of *M. rotundata* nest cues, and likely contributes to cues of *O. lignaria* nests. Other studies have shown the importance and multi-functionality of lipids in the chemical mediation of the behavior of these bees. In a study of nest site attraction (rather than recognition), we discovered that short-chained FFAs (10, 12 and 14 carbons) discovered in solvent extracts of *O. lignaria* cocoons were attractive to nest-seeking females (Pitts-Singer et al. 2016), and application of these FFAs to nesting sites increased the use of those nests by *O. lignaria* females in commercial settings. Also, Paulmier et al. (1999) found that the solvent extract of cuticles of young *M. rotundata* females was attractive to male bees, and used a multivariate analysis to show that relative proportions of alkenes was indicative of young and old females. Managed solitary bees use chemical signals to optimize their ability to mate, nest, and reproduce. Awareness of important bee cues and their functions may help bee managers to make informed decisions and avoid disruption of bee activity, such as using chemicals for cleaning bee nest materials or for applying pesticides and additives (e.g., unknown components of product formulations or adjuvants) that could mask or remove important odors.

### Acknowlegdements

We thank Charlotte Fatland for the many GC-MS analyses and her expertise in identifying lipids. Additionally, a small army of laboratory assistants provided support in the lipid analyses: Tonya Becker-Bolton, Kelly Benson, Emilie Vomhof-DeKrey, Thunyaporn Jeradechachai, Jiyan Mohammed, Robin Schiermeister, and Spencer Nelson. Ellen Klomps helped to collect bees, and Christelle Guédot provided a few bees and performed their dissections in one year. Critical comments from Natalie Boyle greatly improved this manuscript. Mention of trade names or commercial products in this publication is solely for the purpose of providing specific information and does not imply recommendation or endorsement by the U.S. Department of Agriculture.

## Electronic supplementary material

ESM 1 (DOCX 676 kb)
