# Supplementary material for: Comparison of the Chemical Compositions of the Cuticle and Dufour’s Gland of Two Solitary Bee Species from Laboratory and Field Conditions
Source: J Chem Ecol. 2017 May 12;43(5):451–68. doi: 10.1007/s10886-017-0844-x (PMC5487754; doi:10.1007/s10886-017-0844-x)
Supplement: Supplementary file 1 — (DOCX 676 kb) [file 10886_2017_844_MOESM1_ESM.docx]

# **Comparison of the Chemical Compositions of the Cuticle and Dufour’s Gland of Two Solitary Bee Species from Laboratory and Field Conditions**

**Theresa L. Pitts-Singer*^1^, Marcia M. Hagen^2^, Bryan R. Helm^3^, Steven Highland^4^, James S. Buckner^2^, William P. Kemp^2^**

^1^USDA ARS Pollinating Insects Research Unit, Utah State University, Logan, UT, USA 84322

^2^USDA ARS Biosciences Research Laboratory, Red River Valley Agricultural Research Center, Fargo, ND, USA 58102

^3^Biological Sciences Department, North Dakota State University, Fargo, ND, USA 58102

^4^Bureau of Land Management - Mt. Lewis Field Office, 50 Bastian Rd., Battle Mountain, NV USA 89820

# **Supplementary Material**

**Supplementary Table 1** For *Osmia lignaria* and *Megachile rotundata* females collected in multiple years: ages when collected, number of samples (*N/N* = number of cuticles / number of Dufour’s glands), month when samples were collected, and time until samples were extracted and dissected

|  | *Megachile rotundata* | | *Osmia lignaria* | |
| --- | --- | --- | --- | --- |
| Year | Field | Laboratory | Field | Laboratory |
| Ages | 2-4 wk | 3-7 d | 2-7 d | 2-3 d |
| 2003 |  |  | 5/5; June; 5 mo |  |
| 2005 |  |  | 5/5; June; 3 mo |  |
| 2006 |  | 7/5; May/ September; 1 mo |  | 4/4; May; 2 wk |
| 2008 | 10/10; August;  2-10 d | 7/7; August; 4-5 d |  |  |
| 2009 |  |  |  | 7/7; March; 1 d |


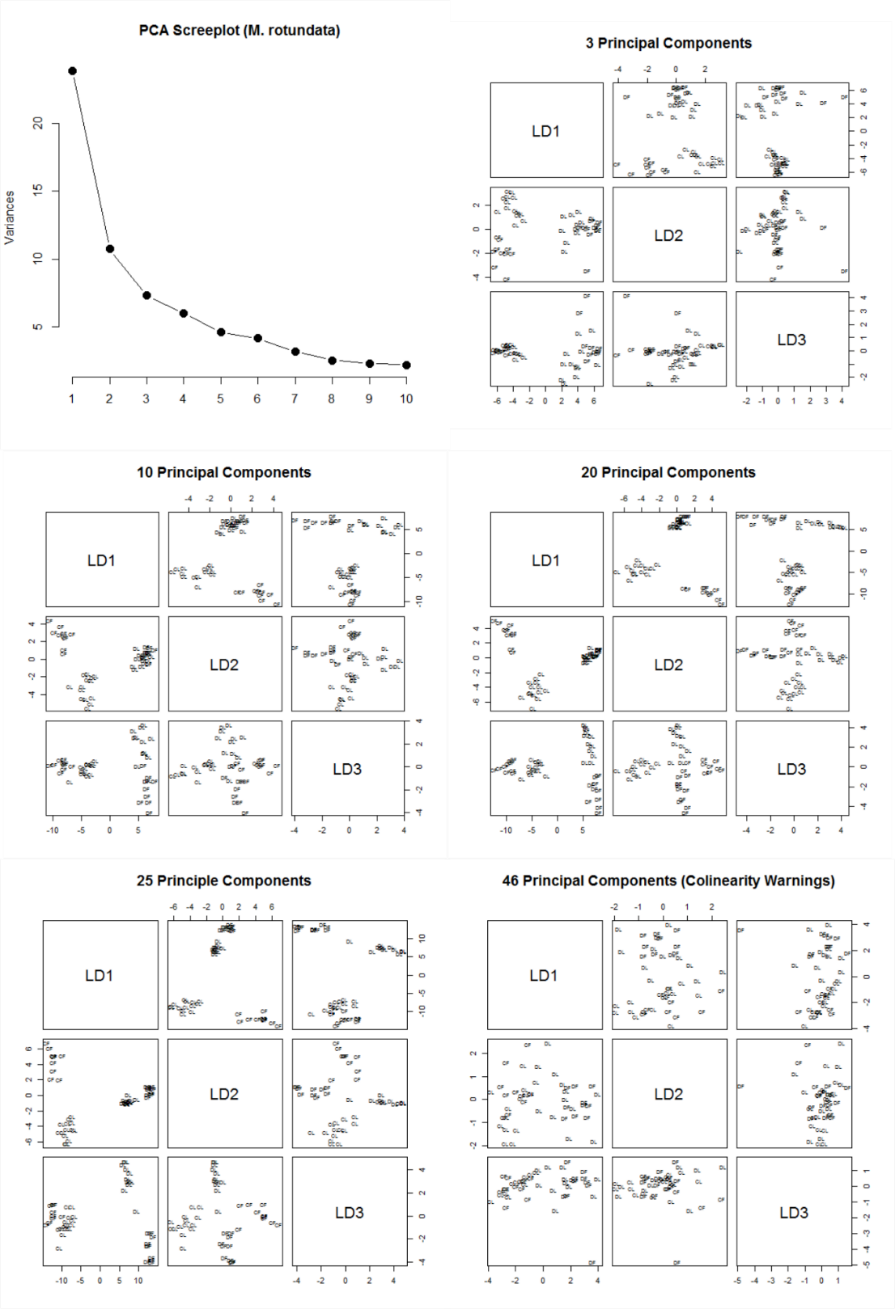


**Supplementary Fig. 1** Screeplot of Principle Components Analysis for *Megachile rotundata* lipids and results of Linear Discriminant Analysis using 3, 10, 20, 25, and 46 principle components, representing 46.6%, 73.3%, 90.2%, 94.8%, and 100% of the variance, respectively. In summary, the general interpretation of LDA results was similar when using different numbers of principle components up to the point where all principle components were used for LDA. However, when using all PCs for LDA, multiple collinearity warnings were flagged by the analysis indicating that these data may not be the best representation of the data


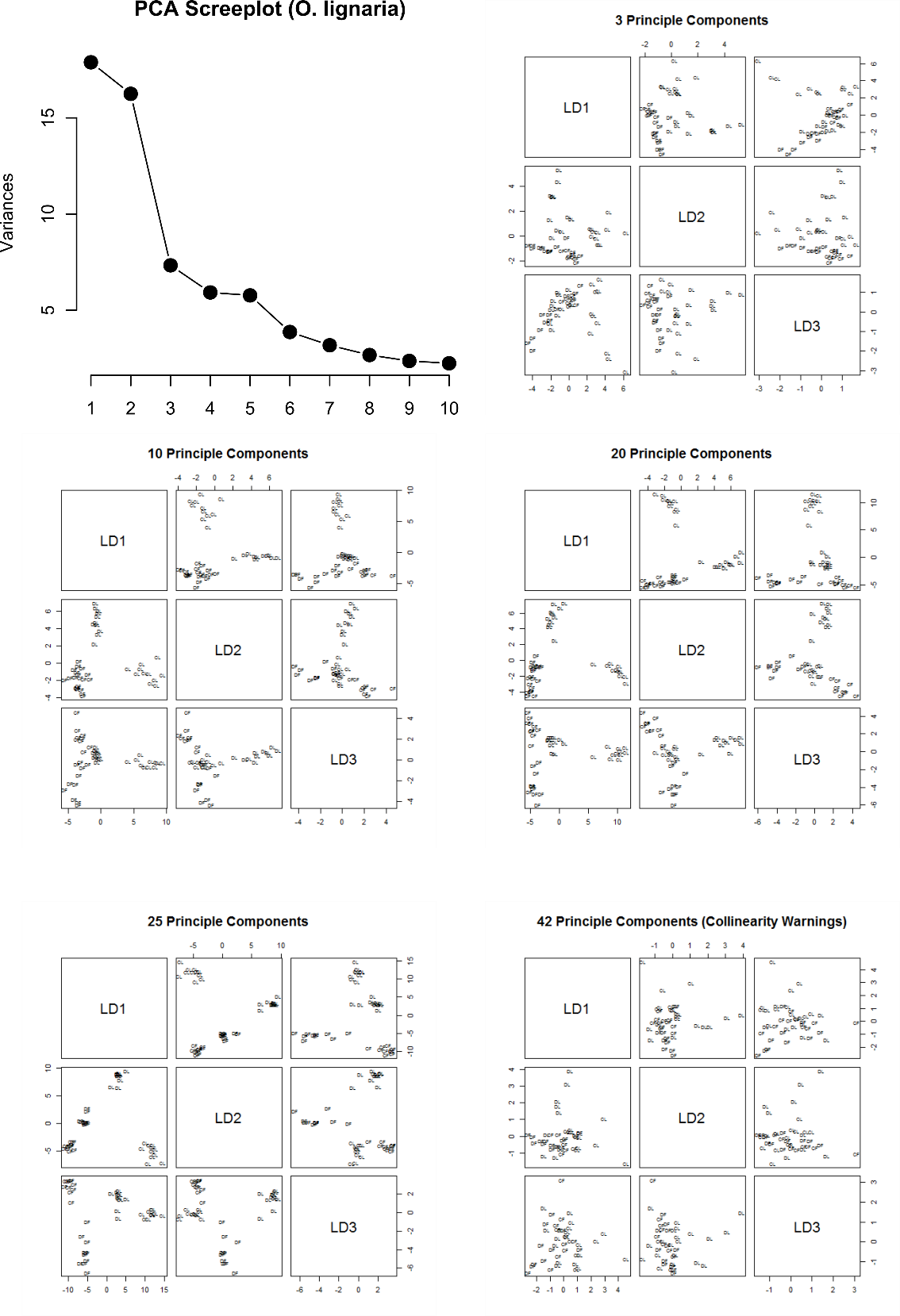


**Supplementary Fig. 2** Screeplot of Principle Components Analysis for *Osmia lignaria* lipid compositions and results of Linear Discriminant Analysis (LDA) using 3, 10, 20, 25, and 46 principle components, representing 48.8%, 79.4%, 93.8%, 97.0%, and 100% of the variance, respectively. In summary, the general interpretation of comparisons from LDA was similar when using different numbers of principle components
